# Food Insecurity and Dietary Deprivation: Migrant Households in Nairobi, Kenya

**DOI:** 10.3390/nu15051215

**Published:** 2023-02-28

**Authors:** Elizabeth Opiyo Onyango, Jonathan S. Crush, Samuel Owuor

**Affiliations:** 1 School of Public Health, University of Alberta, Edmonton, AB T6G 2R3, Canada; 2 Balsillie School of International Affairs, Laurier University, Waterloo, ON N2L 6C2, Canada; 3 University of the Western Cape, Cape Town 7535, South Africa; 4 Department of Geography, Population and Environmental Studies, University of Nairobi, Nairobi P.O. Box 30197-00100, Kenya

**Keywords:** dietary diversity, food security, urban migrants, rural-urban links, internal migration, Nairobi

## Abstract

The current study focuses on food consumption and dietary diversity among internal migrant households in Kenya using data from a city-wide household survey of Nairobi conducted in 2018. The paper examined whether migrant households are more likely to experience inferior diets, low dietary diversity, and increased dietary deprivation than their local counterparts. Second, it assesses whether some migrant households experience greater dietary deprivation than others. Third, it analyses whether rural-urban links play a role in boosting dietary diversity among migrant households. Length of stay in the city, the strength of rural-urban links, and food transfers do not show a significant relationship with greater dietary diversity. Better predictors of whether a household is able to escape dietary deprivation include education, employment, and household income. Food price increases also decrease dietary diversity as migrant households adjust their purchasing and consumption patterns. The analysis shows that food security and dietary diversity have a strong relationship with one another: food insecure households also experience the lowest levels of dietary diversity, and food secure households the highest.

## 1. Introduction

Cities in Sub-Saharan Africa (SSA) are experiencing rapid growth with high rates of natural population increase and rural-to-urban migration [1]. UN-Habitat (2014) estimated that 200 million people in Africa or 62% of the region’s urban population reside in city-centre slums and peripheral informal settlements. Many migrant households in the city live a precarious existence in these underserviced and overcrowded slums and settlements, unable to secure regular or any employment, and incapable of meeting many basic needs [2,3,4,5]. Poor neighbourhoods in most cities are not only deprived of livelihood opportunities and basic amenities, but disproportionally bear the burden of food insecurity [6,7,8]. As a result, slums and informal settlements in African cities have recently been labelled ‘urban food deserts’, reflecting the fact that many residents are both chronically food insecure, and vulnerable to political, economic, and environmental shocks that increase food insecurity [9,10,11,12,13].

The UN Food and Agriculture Organization [14] (p. 107) defines food security as existing when “all people, at all times, have physical, social and economic access to sufficient, safe and nutritious food that meets their dietary needs and food preferences for an active and healthy life.” This definition has four inter-related dimensions—food availability, food accessibility, food utilisation, and food stability. There is a large body of research in SSA on food availability [15,16,17,18] and a growing amount of literature on urban food accessibility; that is, the ability of households to ensure physical, social and economic access to enough nutritious food [6,10,19,20]. Somewhat less attention has been paid to food utilisation under conditions of hyper-urbanisation, although this is changing with the realisation that African cities are undergoing a major nutrition transition and shifts in the urban food environment and diet [21,22,23,24,25,26,27]. To capture the utilisation dimension of food security, dietary diversity is viewed as a robust proxy for the nutritional quality of the diet of individuals and households and the socio-economic status of households [28].

In Kenya, the study of dietary diversity is dominated by research on the relationship between smallholder agricultural production and dietary diversity in rural areas (for example, [29,30,31,32]). Less attention has been given to dietary diversity in urbanising Kenya, although that is beginning to change [33]. Kimani-Murage and colleagues in two separate papers [34,35] showed that slum households are not only highly vulnerable to food insecurity but also experience a double burden of malnutrition with high levels of chronic child malnutrition co-existing with high levels of maternal obesity. A recent household survey in the secondary city of Kisumu showed a clear link between poverty and dietary deprivation [32]. Another study of three small towns in central Kenya found significant negative shifts in dietary composition accompanying supermarket shopping with increased consumption of highly processed foods [33,36,37,38].

In this paper, we focus on the capital city of Kenya, Nairobi, the largest and most important city in the country (with a population of 4.4 million) for four main reasons. First, although there is a growing amount of literature on the levels and determinants of food insecurity in this city, little attention has been paid to the links between migration (which is driving the city’s rapid growth) and the quality of household diets. Second, there is evidence that migrant households in Nairobi retain strong multigenerational links with rural areas, and it is important to assess whether these links play any role in mitigating food insecurity and enhancing dietary diversity in the city [39]. Third, we conducted the first city-wide representative household food security survey in Nairobi in 2018 and are able to freely mine this large dataset for insights on the drivers of contemporary migration and dietary diversity in one of Africa’s most important cities. Finally, the city of Nairobi released an innovative and comprehensive Food System Strategy in March 2022, one of the first of its kind in Africa [40]. Monitoring and evaluation is a central component of this policy initiative, and this paper provides city policy makers with baseline information on dietary deprivation that can later be revisited to evaluate the impacts of the strategy on the quality of household diets in the city.

The paper is a contribution to the growing amount of literature on urban food security in African cities and, in particular, to the emerging interest in the changing quality of food consumption among the newly urbanised. The paper has three main research objectives: First, it examines whether migrant households in Nairobi are more likely to experience inferior diets, low dietary diversity, and increased dietary deprivation. Second, it assesses whether and why some migrant households are more vulnerable to dietary deprivation than others. Third, the paper analyses whether rural-urban links play a role in boosting dietary diversity among migrant households in the city.

## 2. Literature Review

Studies of low-income neighbourhoods in African cities have found consistently low levels of dietary diversity and identified a series of actual and potential variables that help to explain variations in household food consumption. A comparative analysis of dietary diversity in low-income communities in eleven Southern African cities, for example, found that households with young children consumed a limited diversity of food and experienced both short-term and long-term food and nutrition insecurity [8]. Studies of food consumption in South African urban informal settlements have identified low levels of dietary diversity, a heavy dependence on cereals, and a strong association between dietary diversity and household income, poverty, and unemployment [41,42,43,44,45].

Researchers in Accra, Ghana, found that household characteristics with statistically significant associations with dietary diversity included the sex and education level of the household head, income, and food source [46]. In Nigeria, as income increases, diets improve in quantity and quality [47]. In Tanzania, Cockx et al. [48] find that urbanisation is significantly associated with important changes in dietary patterns, including a shift away from traditional staples towards more processed and ready-to-eat foods and heightened consumption of sugar and fats. In Ghana and Cameroon, smaller, better-off, and more educated households with higher levels of dietary diversity are less likely to respond to rising food prices by reducing diets or shifting buying patterns [49].

In urban Africa, households rely on food purchase from the formal or informal sector for the majority of their food needs. This means that they are particularly attuned and sensitive to food price increases. The 2007–2008 global food price crisis, for example, led to a sudden and dramatic increase in urban food insecurity [50,51], as has the more recent COVID-19 pandemic [50,52,53]. While sudden or gradual food price increases led to various coping behaviours such as eating less and eating less often, more research is needed on how food price increases in particular food groups impact food consumption and dietary diversity. An innovative study in Burkina Faso did find that dietary diversity significantly decreased between 2007 and 2008 as food prices increased [12]. While households increased their expenditure on food, it was insufficient to compensate for the effects of the food price crisis earlier.

In Nairobi, most research on food consumption and dietary diversity has targeted the city’s low-income informal slums, home to 60% of the population. These studies have shown that poverty is deeply entwined with food insecurity and dietary deprivation. Poor dietary diversity and associated health outcomes have been attributed to the food environment in these low-income areas of the city [54,55,56]. However, Dominguez-Salas et al. [57] note that, even among low-income households in Nairobi, there can be a wide range of variability in the predictors of positive nutritional status, which include income, household head education, and female headship. Higher education is also likely to be associated with higher knowledge, higher income, and more positive health and nutrition practices. In a separate survey of low-income urban and peri-urban households, Nyakundi et al. [58] posit that households that are income or asset-poor have poor dietary diversity. In addition, they suggest that there is a strong overlap between food insecurity and dietary diversity, with food insecure households having poor dietary diversity.

Several scholars have argued for a greater general focus on the connections between migration and dietary diversity under conditions of rapid urbanisation in the Global South [59,60,61,62]. However, very few studies of dietary diversity in urban Kenya or other African cities identify the migration status of a household as a potential factor influencing dietary diversity. There are even fewer examples of studies focused specifically on migrants as a target group, and who are potentially more vulnerable to dietary deprivation. One study of Zimbabwean migrants in South African cities found that migrants do have higher levels of food insecurity than locals, low levels of dietary diversity, and diets high in starch and excessive concentrations of sugar and oils [11]. Protein and vitamin-rich foods are consumed in only a minority of migrant households [11]. Migrants are more food insecure and have poorer diets than their local counterparts in part because remittances reduce their disposable income.

In relation to internal migration, one study in Uganda found that remitting did not, in fact, reduce the food insecurity of remitters [63]. Pendleton et al. [64] further show that migrant households in Windhoek, Namibia, have a less diverse diet than other households and that a constant complaint is the lack of variety and the monotony of the diet. In Kampala, Uganda, new migrants to the city were found to be more vulnerable than established urban residents to low dietary diversity, and lower-income groups compensate by participating in food sharing networks [65]. Another study of a small urban centre in Zambia suggests that recent migrants have significantly better food access than non-migrant households and those that migrated earlier [66]. The nature and durability of rural-urban links and their role in improving dietary diversity have also received some attention, particularly in relation to informal food transfers from rural households to their urban counterparts [13,15,67].

The varied, and sometimes contradictory, findings of these case studies raise a number of important questions with significant policy implications. For example, are migrant households particularly vulnerable to food insecurity and poor dietary diversity under conditions of rapid urbanisation and in-migration? Does dietary diversity improve or decline with length of residence in the city? Are all migrant households in the city equally able to secure healthy diets and optimal dietary diversity? Is the quality of the diet of migrant households impacted by the strength of their rural-urban links? The inconclusive answers to these questions to date strongly suggest the need for more empirical studies using common methodological approaches before generalisations are possible about the broader connections between migration and dietary deprivation.

## 3. Materials and Methods

### 3.1. Study Design and Participants

The primary objectives of the analysis were threefold: (a) to examine the relationship between household dietary diversity and household characteristics by determining which migrant household demographic and socio-economic variables had a significant relationship with dietary diversity; (b) to assess whether food price changes lead to a decrease in dietary diversity; and (c) to identify which migrant households are most likely to experience low dietary diversity and are therefore most vulnerable to its nutritional and health-related consequences. To address these questions, the paper draws on data from a household food security survey in Nairobi City in 2018 [68]. The survey involved a cross-sectional city-wide representative sample of 1414 households. The number of sampled households was determined using a multi-stage proportional-to-population size random sampling procedure. A three-stage cluster sampling strategy was used to identify 23 sublocations from 8 divisions in the 4 districts/sub-counties of Nairobi City. In the selected 23 sub-locations, systematic random sampling was used to identify the participating households, where every n^th^ household was recruited and interviewed. The household head was the target interviewee in this survey. The data were collected in face-to-face interviews by trained enumerators using tablets for data collection. For the analysis in this paper, we drew a sub-sample of 941 (67%) migrant households defined as households with a head who was an internal migrant who originated from another part of the country.

### 3.2. Data Collection

The survey instrument collected information on household and individual demographic characteristics, the social and economic profile of the households (including employment, income, and expenditure), the health status of household members, household food consumption, and sources of food including formal, informal, and non-market sources. Data were also collected on household experiences of food price changes, change in the price of foods in specific food groups, and the effects of the food price change on household food consumption patterns.

#### 3.2.1. Dependent Variables

The most widely used validated household dietary diversity metric is the Household Dietary Diversity Score (HDDS) [69,70,71]. Data on household intake of foods from 12 food groups are collected for a 24-h recall. This study used the FAO standard classification of food groups (Table 1). Values for each food group were assigned “0” and “1”, where “0” equals not consumed and “1” equals consumed. An HDDS score was calculated for each household on a scale from 0 to 12, where higher scores indicate greater dietary diversity. For this analysis, the HDDS scale was binned to create four categorical variables.

Household food security was measured using the Household Food Insecurity Access Score (HFIAS) [72]. An HFIAS score was first calculated for each household based on answers to nine frequency of occurrence questions in the previous four weeks (Table 2). Scores range between 0 and 27, with a score of 0 indicating that the household is completely food secure, and a maximum score of 27 indicating extreme food insecurity. The HFIAS responses are converted into a categorical variable, the HFIAP, using an algorithm to generate a four-part classification—food secure, mildly food insecure, moderately food insecure, and severely food insecure [72,73].

#### 3.2.2. Independent Variables

The dependent and independent variables used in the analysis are summarised in Table 3. The independent variables include the sex, age, education, employment status, and health status of the household head at the time of the survey. The health status variable is a self-reported binary response to questions about whether the household head had any diagnosed health issues from a list including NCDs and communicable disease. Migration history is an ordinal variable designed to capture the time elapsed since the household head first migrated to Nairobi. Household variables in the analysis include household size, household type, house structure, main source of income, total monthly income, health status, and proportion of income spent on food. Households were grouped into four types: female-centred (a female head with no spouse/partner plus child dependents); male-centred (a male head with no spouse/partner plus child dependents); nuclear (two parents plus child dependents); and extended (two parents plus child dependents plus other relatives and non-relatives).

Housing structure is a binary observational response (formal/informal), which was preferred as a formal/informal settlement binary, since the latter contains both formal and informal housing structures in Nairobi. The Lived Poverty Index (LPI) is a validated self-assessment tool for measuring the subjective experience of poverty based on the frequency with which households go without certain basic needs (food, water, medical care, cooking fuel, and cash income) [74]. Other binary household variables include whether or not a household remits cash to the rural areas, receives food transfers from the countryside, participates in urban agriculture, shops for food at supermarkets, and shares meals with neighbours, all of which have the potential to affect dietary diversity.

Onyango et al. [39] show that sudden economic shocks including sharp food price increases have a significant impact on low-income Nairobi households. A key issue is whether food price volatility in different food groups is associated with reduced dietary diversity. The food price volatility variable in this analysis is based on questions about whether or not food price increases had negatively impacted the household in the six months prior to the survey. This measure was an ordinal variable with four options: never, about once a month, about once a week, or nearly every day of the week. Because food price volatility tends to affect some marketed foods more than others, an additional 11 binary variables were generated relating to which foods had become unaffordable.

### 3.3. Data Analysis

The data were analysed using the software SPSS version 28 (IBM Statistics 28). The analysis included descriptive percentages, bar graphs, and crosstabulation to provide an overview of the distribution of response variables and the food security and dietary diversity frequency distributions. To investigate the relationship between the dependent and independent variables, we use logistic regression modelling in the form of ordinal logit analysis. A chi-square test was performed to determine within and between-group differences for the explanatory and response variables. Ordinal regression modelling was performed to ascertain which household characteristics and food price changes were associated with household dietary diversity and for the generation of the exponentials (odds ratios). The ordinal cumulative logit link was used given the ordered nature of the dependent HDDS variable.

## 4. Results

### 4.1. Household Characteristics

Table 4 summarises the socio-demographic and economic characteristics of the sampled migrant households. The majority of the household heads are male (82.5%), with only 17.5% headed by females. Most migrant heads (80%) are of working age, between 25 and 55 years. The small proportion of migrant heads over the age of 55 is a reflection of the fact that retirees in Nairobi tend to return to their rural homes [75]. The household heads are relatively well-educated, with over 80% having some secondary or tertiary education. Less than 1% have no education. Almost all migrant household heads in Nairobi have some form of employment, although only 42% are employed full-time in the formal sector. Nearly 40% are self-employed, mainly in the informal sector, and only 4% report that they are currently unemployed.

Migrant households vary considerably in size and type. Only 17% are single-person households, while 45% have four or more household members. Female-centred households make up 17% of the total, and male-centred another 20%. Over half of the households are nuclear families, and only a small number are extended families. Nine in ten of the households live in formal dwellings in informal and formal areas. The main source of income for nearly half of the households is formal employment, followed by informal employment (30%) and self-employment (23%). Household incomes also vary considerably, with half of the households making KShs 20,000 (USD 200 or less) per month and nearly 30% with incomes of over KShs 40,000 (USD 400) per month. The Lived Poverty Index is more concentrated with two-thirds of households scoring between 0 and 0.5.

The proportion of income that a household spends on food is a common poverty proxy. This is generally high among migrant households in Nairobi, with 65% spending more than a third of their income on food, and 44% spending more than half on food. Around half of all migrant households send cash remittances to rural areas. In relation to food sourcing, most households (90%) do not engage in meal sharing with neighbouring households and only 2% engage in urban agriculture, which is often held out by advocates as a key to greater food security and dietary diversity [76,77]. Meanwhile, more than half receive food remittances from the rural areas, which could mean greater dietary diversity. Finally, three-quarters of migrant households purchase at least some of their food from supermarkets in Nairobi. Around 60% of migrant households said they had sacrificed eating some foods due to food price increases in the six months prior to the survey. Nearly 40% of households experienced this at least once a week, or more frequently still. The food groups most severely impacted were cereals/grain (affecting 27% of households), and red meat, poultry, and offal (affecting 43% of households).

### 4.2. Household Food Security and Dietary Diversity

Table 5 shows that only 26% of the surveyed migrant households were completely food secure on the HFIAP scale. All of the other households experienced some degree of food insecurity, with 27% experiencing severe food insecurity, 35% moderate food insecurity, and 12% mild food insecurity. On the HDDS, 11% of migrant households consumed food from only 1–3 food groups, indicating an extremely deprived and monotonous diet. As many as half of the households had scores between 4 and 6, with milder dietary deprivation. The remaining 38% had scores of 7 or more, indicative of a more balanced and diverse diet.

**Figure 1:**
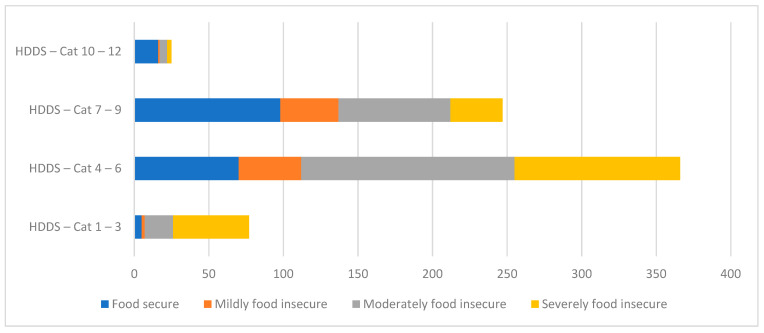
Relationship between household dietary diversity and food security. Notes: HDDS = Household Dietary Diversity Score; Cat = Category.

Table 6 cross-tabulates the HFIAP and HDDS scores to assess whether there is a relationship between food insecurity and dietary diversity. Two-thirds of households with the lowest dietary diversity (HDDS 1–3) are also severely food insecure on the HFIAP. As dietary diversity increases, severe food insecurity declines to 30% (for HDDS 4–6), 14% (for HDDS 7–9), and 12% (HDDS 10–12). The reverse pattern is true for food security, which increases from 6.5% of households in HDDS 0–3, to 19% (HDDS 4–6), 40% (HDDS 7–9), and 64% (HDDS 10–12). Figure 1 confirms that the majority of severely food-insecure migrant households fall into the two lowest dietary diversity categories (HDDS 1–3 and 4–6).

### 4.3. Household Characteristics and Dietary Diversity

Table 7 cross-tabulates dietary diversity with household variables to identify any relationships of potential significance. The *p*-values for the variables in the table provide a first approximation of the relationships of statistical significance. While the HDDS distribution does vary with household head characteristics including sex, age, and health status, the significance of the relationship is very weak. Far more significant is the education level and employment status of the household head. As educational attainment increases, so does dietary diversity. Employment status is a good predictor of dietary diversity; households with heads in full-time wage employment have the highest dietary diversity, followed by those in self-employment. Households with unemployed heads have the lowest dietary diversity.

In regards to household characteristics, the distribution of HDDS scores is weakly related to migrant household size, household type, and the health of household members. Dietary diversity is strongly related to household economic variables. Households in formal housing, with formal wages as the main source of income, and lowest lived poverty all have significantly greater dietary diversity. The relationship between dietary diversity and household income as well as the proportion of income spent on food is statistically significant. For example, 75% of households in the lowest income sextile are in the two lowest HDDS categories, compared with only 34% in the highest sextile. The only other variable with a statistically significant relationship with dietary diversity is supermarket patronage, which tends to be associated with increased diversity.

**Figure 2:**
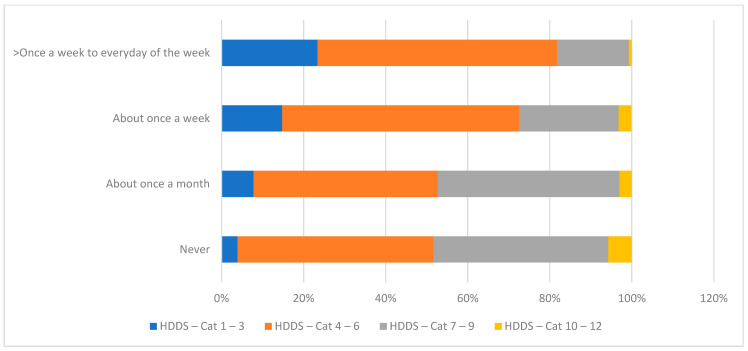
Dietary diversity and frequency of food price increases. Notes: HDDS = Household Dietary Diversity Score; Cat = Category.

Increased food prices are a key determinant of what lower-income households can afford and the kind of foods that end up on the dining table in urban centres. Of the surveyed migrant households, 62% had gone without certain foods due to increases in food prices. The frequency with which they adjusted their food consumption was a significant determinant of dietary diversity. For example, households that experienced food price increases almost every day of the week represented over 50% of those with lower food diversity (Figure 2). Foods most often sacrificed due to price increases included staple grains, meat, eggs, dairy, fish, and, to a lesser extent, vegetables.

### 4.4. Predictors of Dietary Diversity

The results of the ordinal logistic regression analysis of predictors of HDDS are presented in Table 8. The table provides the predictive odds ratios (OR) for the explanatory variables together with the accompanying 95% CI and the level of significance. The education level and employment status of the migrant household head are confirmed as significant predictors of HDDS. Each successively higher level of education is associated with increased odds of higher dietary diversity (OR:1.54 (95% CI of 1.212–1.944)). The employment status of the household head was associated with a lower odds ratio, meaning that households with an unemployed household head had reduced odds of dietary diversity compared with households with a head in full-time wage employment or self-employment (OR: 0.770 (95%CI = 0.646–0.919)). Female heads had higher odds than male heads of belonging to households with greater dietary diversity (OR: 1.203 (95%CI = 0.669–2.163)). Although the age of the household head is an insignificant predictor of dietary diversity, it does have a positive predictive relationship (OR: 1.085 (95%CI = 0.923–1.274)), suggesting that households with older heads have slightly higher odds of having greater dietary diversity.

Various household characteristics were also good predictors of whether a household would have higher dietary diversity. The most significant was housing type; households in formal dwellings had increased odds of better dietary diversity compared to those in informal dwellings (OR: 1.273 (95%CI = 0.825–1.966)). A number of other household characteristics were not significant predictors of dietary diversity. These included household size (OR: 0.978 (95%CI = 0.827–1.157)), household structure (OR: 0.942 (95%CI = 0.706–1.257)), and household health status (OR: 0.884 (95%CI = 0.538–1.450)). Households with more members, those experiencing some health issues, and those with male heads have slightly reduced odds of dietary diversity. However, household socioeconomic factors such as monthly income and lived poverty were significant predictors of migrant household diet diversity. Households with a higher monthly income were more likely to have higher dietary diversity (OR: 1.108 (95%CI = 0.983–1.249)). Households that reported a higher LPI (i.e., greater poverty) had lower odds of dietary diversity (OR: 0.792 (0.647–0.970)).

Sharing meals with neighbours was associated with lower odds of dietary diversity, as these were more likely to be poorer households as well (OR: 0.895 (95%CI = 0.544–1.474)). On the other hand, purchase of food from supermarkets was associated with higher odds of dietary diversity (OR: 1.235 (95%CI = 0.850–1.793)). Few households participate in urban agriculture, and those that did grow some of their own food did not have increased odds of dietary diversity (OR: 0.927 (95%CI = 0.295–2.913)). Similarly, the proportion of income that a household spent on food was not a significant predictor of dietary diversity. Although statistically insignificant, going without certain foods due to food price increases was associated with reduced odds of dietary diversity (OR: 0.941 (95%CI = 0.763–1.161)). However, going without both fruits and vegetables due to food price increases was actually associated with increased dietary diversity (OR: 2.098 (95%CI = 1.238–3.555) and OR: 1.790 (1.001–3.340)). This might be because respondents who considered fruits and vegetable an important part of their diet were of higher socioeconomic status and increases in the price of these foods did not reduce their dietary diversity scores.

Finally, the predictive relationship between two different indicators of household food security with different metrics and recall periods is of interest. In theory, we might expect the HFIAP and HDDS to move in tandem. As food security increases, so does dietary diversity, and vice-versa. This was confirmed by the negative predictive relationship between the two; that is, as food security declined, so did the odds of a household experiencing greater dietary diversity (OR: 0.760 (95%CI = 0.630–0.917)).

## 5. Discussion

In this paper, we set out to examine the food security experience of Nairobi households that trace their origins to the rural areas of Kenya and with which many retain strong connections [13,75]. To identify migrant households in a larger representative household survey dataset, we used the criterion of whether the household head had been born elsewhere in the country. Using this criterion, two-thirds of all households in the Nairobi dataset qualified for inclusion. The sub-sample of 941 households was sufficiently large and representative to draw conclusions about the migrant population of the city. The most obvious feature of the population is its demographic and socio-economic diversity. In part, this is a function of the long history of post-independence migration to the city. Nearly a quarter of the household heads had first migrated to Nairobi over 20 years previously and 45% had been living in Nairobi for over 15 years. Only sixteen percent were relatively recent migrants, many in one-person households (that made up 17% of the total). One sign of the ongoing strength of rural connections is that around half had sent cash remittances in the previous year and half had received food remittances from rural relatives, most on a monthly basis [39].

Most of the household heads (over 80%) were male in three types of households—male-centred, nuclear, and extended. The only households with female heads were also female-centred without a male spouse or partner (17% of the total). Only half of the households had a household head or member in full-time formal sector employment. The majority of other households obtained their main source of income from informal employment or self-employment (42% combined). As a result, there was significant income spread, with 50% earning less than KES 20,000 per month and 30% earning more than twice that amount. Very few migrant households in Nairobi participate in urban agriculture, with lack of access to land for cultivation in the city a primary constraint [68]. Almost all migrant households are therefore reliant on the market for most of their food supply, and well over half spend greater than 35% of household income on food purchase.

With regard to the first objective of the analysis, migrant households in Nairobi do have higher levels of food insecurity on average than non-migrant households, with only 25% of migrant households completely food secure on the HFIAP scale, compared to 35% of non-migrant households [39]. The primary focus of this paper was not on levels of food security per se, but on the quality of household diets as measured by the HDDS. However, as Figure 1 clearly shows, there is a strong association between food security and dietary diversity among migrant households in Nairobi: as food security increases, so does dietary diversity, and, as food insecurity increases, dietary diversity declines.

As expected, increased food prices, which made food less affordable, had a strong positive association with dietary deprivation. Household dietary diversity varied significantly with price increases in most food groups. As the frequency of being affected increased, dietary diversity declined. Virtually every affected food group (with the exception of roots and tubers, fruit, and oils and fats) had a statistically significant relationship with household dietary diversity. Further insights into this relationship were provided by the cross-tabulation of HDDS scores with price increases in particular food groups. Staple foods essential to a balanced and nutritious diet were all affected.

The second objective of the paper was to investigate whether all migrant households experience similar levels of food insecurity and dietary deprivation in Nairobi. Here, the analysis clearly revealed considerable variability. Table 5 shows that 27% were severely food insecure, 47% were mildly or moderately food insecure, and 26% were food secure. Additionally, 11% experienced severe dietary deprivation (HDDS 1–3), 51% moderate deprivation (HDDS 4–6), and 38% little or no dietary deprivation (HDDS 7–12). Table 6 shows that food insecurity and dietary deprivation are closely associated. For example, households with the highest levels of dietary deprivation are also the most food insecure. As dietary diversity increases, food insecurity declines.

The two household head variables with a statistically significant relationship to dietary diversity were their amount of education and employment status. Both the age of the household head and their migration history had a weak relationship with dietary diversity. Dietary diversity, therefore, appears to be largely unrelated to the length of time since initial migration to the city. Economic variables that had the strongest statistical relationship with dietary diversity at household level included the main source of household income (formal or informal, full-time or part-time), household monthly income, lived poverty, the amount of household income spent on food, and whether the household lives in formal or informal housing.

The ordinal logistic regression analysis of predictors of dietary diversity provided a more robust analysis of the odds of a household experiencing dietary deprivation. Migrant households with poorly educated heads without full-time employment had the highest odds of dietary deprivation. Male heads had slightly lower odds than female heads of belonging to households with low dietary diversity. Although migrant female-headed households were more likely to be food insecure overall than male-headed households, the reverse was true with regard to dietary diversity. This suggests that female heads are more likely to husband their resources to ensure a more balanced diet for household members [13,47]. Other household characteristics associated with increased odds of dietary deprivation included residence in informal housing, low monthly income, and increased lived poverty.

The third objective of the paper was to analyse whether the strength of rural-urban links affects dietary diversity among migrant households in Nairobi. This analysis did not find that the degree of dietary diversity had a strong relationship with the duration of residence of the migrant household head in the city. Length of time since migration did not mean increased or decreased odds of dietary deprivation. Based on studies elsewhere, cash remitting to the rural areas was expected to decrease dietary diversity by reducing the migrant household’s disposable income [2]. On the other hand, informal food transfers were expected to increase dietary diversity by augmenting the household food supply with fresh produce from the countryside [19]. The analysis confirmed that migrant households that remit are no more or less likely to experience dietary deprivation. In addition, those that receive food transfers are no more likely than those that do not to have greater dietary diversity. This has potentially important policy implications in light of recent policy interventions focused on making rural-urban connections in Africa more robust. While these policies may offer some benefit to rural and urban dwellers, this case study suggests that improvement in dietary diversity is not one of them.

## 6. Conclusions

This paper demonstrates that many migrant households across the city are vulnerable to food insecurity and dietary deprivation. Three-quarters of Nairobi’s migrant households experience some degree of food insecurity and almost two-thirds experience a degree of dietary deprivation. Further, there is a clear relationship between food insecurity and dietary deprivation; that is, food insecure households are also much more likely to experience low levels of dietary diversity. This reciprocal relationship means that measures to reduce food insecurity by increasing food accessibility are also likely to improve dietary diversity and that strategies to deal with dietary deprivation, such as food fortification, will impact positively on food insecurity. Despite the high levels of food insecurity and dietary deprivation amongst migrant households, not all are equally vulnerable. While the determinants of this distribution are complex and challenging to decipher given the cross-sectional nature of the data, the survey results identified that those households most likely to be associated with dietary deprivation have low incomes, high levels of lived poverty, and limited access to formal sector wage employment.

Regarding the more general implications of the study, acculturation theory in the Global North asserts that the nutritional quality of migrant food consumption tends to decline over time [78,79,80,81,82]. Studies of this phenomenon in relation to migration in the Global South are less common and the limited evidence that exists is inconclusive [83,84,85]. One hypothesis in line with acculturation theory is that migrants to the city in the South experience a decline in dietary quality over time, as they consume less fresh produce and more processed food. An alternative position is that new migrants are more likely to experience immediate dietary deprivation, but that, over time, as they gain greater access to livelihood opportunities and build social and economic support networks in the city, dietary diversity improves. The evidence from Nairobi does not give strong support for either position. Both the descriptive statistics and the ordinal logistic regression indicate that dietary diversity is unrelated to the passage of time since the household head first migrated to Nairobi. The prevalence of dietary deprivation is very similar for short, medium, and long-term migrants in the city. This suggests that the theory from the Global North is inappropriate for the dietary experience of migrants within the Global South, and that alternative theory-building more appropriate to Southern realities is necessary.

## Figures and Tables

**Table 1:** HDDS food groups.

1	Cereals	0 = not consumed, 1 = consumed
2	Roots and tubers	0 = not consumed, 1 = consumed
3	Vegetables	0 = not consumed, 1 = consumed
4	Fruits	0 = not consumed, 1 = consumed
5	Meat, poultry, offal	0 = not consumed, 1 = consumed
6	Eggs	0 = not consumed, 1 = consumed
7	Fish and seafood	0 = not consumed, 1 = consumed
8	Pulses, legumes, and nuts	0 = not consumed, 1 = consumed
9	Milk and milk products	0 = not consumed, 1 = consumed
10	Oils/fats	0 = not consumed, 1 = consumed
11	Sugar/honey	0 = not consumed, 1 = consumed
12	Miscellaneous	0 = not consumed, 1 = consumed

**Table 2:** HFIAS questions.

Questions	Frequency
1: In the past four weeks, did you worry that your household would not have enough food?	0 = No, 1 = Rarely, 2 = Sometimes, 3 = Often
2: In the past four weeks, were you or any household member not able to eat the kinds of foods you preferred because of a lack of resources?	0 = No, 1 = Rarely, 2 = Sometimes, 3 = Often
3: In the past four weeks, did you or any household member have to eat a limited variety of foods due to a lack of resources?	0 = No, 1 = Rarely, 2 = Sometimes, 3 = Often
4: In the past four weeks, did you or any household member have to eat some foods that you really did not want to eat because of a lack of resources to obtain other types of food?	0 = No, 1 = Rarely, 2 = Sometimes, 3 = Often
5: In the past four weeks, did you or any household member have to eat a smaller meal than you felt you needed because there was not enough food?	0 = No, 1 = Rarely, 2 = Sometimes, 3 = Often
6: In the past four weeks, did you or any household member have to eat fewer meals in a day because there was not enough food?	0 = No, 1 = Rarely, 2 = Sometimes, 3 = Often
7: In the past four weeks, was there ever no food to eat of any kind in your household because of lack of resources to get food?	0 = No, 1 = Rarely, 2 = Sometimes, 3 = Often
8: In the past four weeks, did you or any household member go to sleep at night hungry because there was not enough food?	0 = No, 1 = Rarely, 2 = Sometimes, 3 = Often
9: In the past four weeks, did you or any household member go a whole day and night without eating anything because there was not enough food?	0 = No, 1 = Rarely, 2 = Sometimes, 3 = Often

**Table 3:** Variables used in analysis.

Dependent Variables	Code
Household Dietary Diversity Score	
HDDS 1–3	1
HDDS 4–6	2
HDDS 7–9	3
HDDS 10–12	4
Household Food Insecurity Access Prevalence (HFIAP)	
Food secure	1
Mildly food insecure	2
Moderately food insecure	3
Severely food insecure	4
Independent Variables	
Sex of Household Head	
Male	1
Female	2
Age of Household Head	
16–24	1
25–34	2
35–44	3
45–54	4
55–64	5
65+	6
Education Level of Household Head	
None	1
Primary school	2
Secondary school	3
Higher	4
Employment Status of Household Head	
Self-employed	1
Employed full-time	2
Employed part-time (incl. casual)	3
Unemployed	4
Duration of Stay in Nairobi City	
≤5 years	1
6–10 years	2
11–15 years	3
16–20 years	4
>20	5
Health Status of Household Head	
Healthy	1
Unhealthy	2
Household Characteristics	
Household Size	
1 person	1
2–3 persons	2
4–5 persons	3
6+ persons	4
Illness in Household	
Yes	1
No	2
Dwelling Type	
Formal	1
Informal	2
Household Structure Type	
Female-centred	1
Male-centred	2
Nuclear	3
Extended	4
Main Source of HH Income	
Formal wage work	1
Informal wage work	2
Self-employment (informal)	3
Self-employment (formal)	4
Total HH Income	
KES ≤ 10,000	1
KES 10,001–20,000	2
KES 20,001–30,000	3
KES 30,001–40,000	4
KES 40,001–50,000	5
>KES 50,000	6
Lived Poverty Index Categories	
0–0.5	1
0.51–1.00	2
1.01–1.50	3
>1.50	4
% of HH Income Spent on Food	
<20%	1
21–35%	2
36–50%	3
>50%	4
Sent Remittances	
Yes	1
No	2
Received Food Transfers from Rural Areas	
Yes	1
No	2
Household Shares Meal with Neighbours	
Yes	1
No	2
Household Purchases Food from Supermarkets	
Yes	1
No	2
Household Engages in Urban Agriculture	
Yes	1
No	2
Gone without Certain Foods due to Food Price Increase	
Never	1
About once a month	2
About once a week	3
More than once a week to every day of the week	4
Gone without Cereals/Grains due to Price Increase	
Yes	1
No	2
Gone without Roots and Tubers due to Price Increase	
Yes	1
No	2
Gone without Vegetables due to Price Increase	
Yes	1
No	2
Gone without Fruits due to Price Increase	
Yes	1
No	2
Gone without Meat, Poultry, or Offal due to Price Increase	
Yes	1
No	2
Gone without Eggs due to Price Increase	
Yes	1
No	2
Gone without Fish and Seafood due to Price Increase	
Yes	1
No	2
Gone without Pulses/Legumes/Nuts due to Price Increase	
Yes	1
No	2
Gone without Milk and Milk Products due to Price Increase	
Yes	1
No	2
Gone without Oils/Fats due to Price Increase	
Yes	1
No	2
Gone without Sugar/Honey due to Price Increase	
Yes	1
No	2

**Table 4:** Migrant household characteristics.

	Frequency	Percentages
Sex of Household Head		
Male	769	82.5
Female	163	17.5
Age of Household Head		
16–24	70	7.5
25–34	318	34.0
35–44	298	31.8
45–54	129	13.8
55–64	53	5.7
65+	68	7.3
Education Level of Household Head		
None	7	0.8
Primary school	158	17.3
Secondary school	369	40.3
Higher	381	41.6
Employment Status of Household Head		
Self-employed	353	38.8
Employed full-time	384	42.2
Employed part-time (incl. casual)	136	14.9
Unemployed	37	4.1
Duration of Stay in Nairobi City		
≤5 years	140	15.7
6–10 years	214	24.0
11–15 years	139	15.6
16–20 years	185	20.8
>20	212	23.8
Health Status of Household Head		
Healthy	875	94.5
Unhealthy	51	5.5
Household Characteristics		
Household Size		
1 person	161	17.2
2–3 persons	337	35.9
4–5 persons	322	34.3
6+ persons	118	12.6
Illness in Household		
Yes	157	16.7
No	784	83.3
Dwelling Type		
Formal	816	90.6
Informal	85	9.4
Household Structure Type		
Female-centred	155	16.6
Male-centred	187	20.0
Nuclear	520	55.7
Extended	71	7.6
Main Source of HH Income		
Formal wage work	440	47.3
Informal wage work	277	29.8
Self-employment (Informal)	110	11.8
Self-employment (formal)	103	11.1
Total HH Income		
KES ≤ 10,000	142	25.1
KES 10,001–20,000	142	25.1
KES 20,001–30,000	75	13.3
KES 30,001–40,000	41	7.3
KES 40,001–50,000	29	5.1
>KES 50,000	136	24.1
Lived Poverty Index Categories		
0–0.5	609	66.3
0.51–1.00	189	20.6
1.01–1.50	77	8.4
>1.50	43	4.7
% of HH Income Spent on Food		
<20%	96	17.5
21–35%	95	17.3
36–50%	117	21.4
>50%	239	43.6
Sent Remittances		
Yes	495	53.9
No	424	46.1
Received Food Transfers from Rural Areas		
Yes	484	52.1
No	445	47.9
Household Shares Meal with Neighbours		
Yes	96	10.2
No	845	89.8
Household Purchases Food from Supermarkets		
Yes	734	78.0
No	207	22.0
Household Engages in Urban Agriculture		
Yes	22	2.3
No	919	97.7
Gone without Certain Foods due to Food Price Increase		
Never	356	38.0
About once a month	229	24.5
About once a week	163	17.4
More than once a week to every day of the week	188	20.1
Gone without Cereals/Grains due to Price Increase		
Yes	255	27.1
No	686	72.9
Gone without Roots and Tubers due to Price Increase		
Yes	116	12.3
No	825	87.7
Gone without Vegetables due to Price Increase		
Yes	76	8.1
No	865	91.9
Gone without Fruits due to Price Increase		
Yes	103	10.9
No	838	89.1
Gone without Meat, Poultry, or Offal due to Price Increase		
Yes	405	43.0
No	536	57.0
Gone without Eggs due to Price Increase		
Yes	72	7.7
No	869	92.3
Gone without Fish and Seafood due to Price Increase		
Yes	308	32.7
No	633	67.3
Gone without Pulses/Legumes/Nuts due to Price Increase		
Yes	61	6.5
No	880	93.5
Gone without Milk and Milk Products due to Price Increase		
Yes	142	15.1
No	799	84.9
Gone without Oils/Fats due to Price Increase		
Yes	77	8.2
No	864	91.8
Gone without Sugar/Honey due to Price Increase		
Yes	114	12.1
No	827	87.9

**Table 5:** Migrant household food security and dietary diversity.

	Frequency	Percent
Food Security (HFIAP)		
Food secure	242	25.8
Mildly food insecure	110	11.7
Moderately food insecure	332	35.4
Severely food insecure	254	27.1
Dietary Diversity (HDDS)		
1–3	77	10.7
4–6	366	51.0
7–9	249	34.7
10–12	26	3.6

**Table 6:** Relationship between migrant household food security and dietary diversity.

Food Insecurity (HFIAP)	HDDS (1–3)	HDDS (4–6)	HDDS (7–9)	HDDS (10–12)
Food secure	5 (6.5)	70 (19.1)	98 (39.7)	16 (64.0)
Mildly food insecure	2 (2.6)	42 (11.5)	39 (15.8)	1 (4.0)
Moderately food insecure	19 (24.7)	143 (39.1)	75 (30.4)	5 (20.0)
Severely food insecure	51 (66.2)	111 (30.3)	35 (14.2)	3 (12.0)
	77 (10.8)	366 (51.2)	247 (34.5)	25 (3.5)

**Table 7:** Migrant household characteristics and dietary diversity.

Migrant-Headed Households	Household Diet Diversity Score (HDDS) Categories				*p*-Value
Characteristics of Migrant Household Heads	1–3	4–6	7–9	10–12	
Sex of Household Head					
Male	58 (10.1)	294 (51.3)	197 (34.4)	24 (4.2)	0.153
Female	19 (13.7)	69 (49.6)	50 (36.0)	1 (0.7)	
Age of Household Head					
16–24	7 (12.3)	27 (47.4)	22 (38.6)	1 (1.8)	0.776
25–34	29 (12.2)	126 (53.2)	72 (30.4)	10 (4.2)	
35–44	21 (9.5)	112 (50.9)	78 (35.5)	9 (4.1)	
45–54	7 (7.4)	47 (49.5)	37 (38.9)	4 (4.2)	
55–64	3 (6.5)	23 (50.03)	18 (39.1)	2 (4.3)	
65+	10 (16.4)	29 (47.5)	22 (36.1)	0 (0.0)	
Education Level of Household Head					
None	1 (14.3)	5 (71.4)	1 (14.3)	0 (0.0)	<0.001
Primary school	29 (22.0)	68 (51.5)	33 (25.0)	2 (1.5)	
Secondary school	42 (14.7)	159 (55.6)	79 (27.6)	6 (2.1)	
Higher	5 (1.9)	120 (44.4)	128 (47.4)	17 (6.3)	
Employment Status of Household Head					
Self-employed	27 (9.7)	135 (48.6)	109 (39.2)	7 (2.5)	<0.001
Employed full-time	16 (5.8)	144 (52.2)	101 (36.6)	15 (5.4)	
Employed part-time (incl. casual)	24 (22.6)	57 (53.8)	24 (22.6)	1 (0.9)	
Unemployed	8 (22.9)	21 (60.0)	6 (17.1)	0	
Duration of Stay in Nairobi City					
≤5 years	13 (11.9)	61 (56.0)	31 (28.4)	4 (3.7)	0.441
6–10 years	13 (8.0)	89 (54.9)	54 (33.3)	6 (3.7)	
11–15 years	17 (17.2)	43 (43.4)	34 (34.3)	5 (5.1)	
16–20 years	11 (8.0)	72 (52.2)	52 (37.7)	3 (2.2)	
>20	18 (11.1)	77 (47.5)	60 (37.0)	7 (4.3)	
Health Status of HH Head					
Healthy	72 (10.8)	343 (51.3)	230 (34.4)	24 (3.60)	0.887
Unhealthy	5 (12.2)	19 (46.3)	16 (39.0)	1 (2.4)	
Household Characteristics					
Household Size					
1 person	14 (11.5)	66 (54.1)	37 (30.3)	5 (4.1)	0.958
2–3 persons	24 (9.3)	129 (50.2)	96 (37.4)	8 (3.1)	
4–5 persons	30 (12.2)	124 (50.6)	82 (33.5)	9 (3.7)	
6+ persons	9 (9.8)	46 (50.0)	33 (35.9)	4 (4.3)	
Illness in HH					
No	59 (9.9)	313 (52.6)	203 (34.1)	20 (3.4)	0.180
Yes	18 (14.6)	53 (43.1)	46 (37.4)	6 (4.9)	
Dwelling Type					
Formal	62 (10.0)	311 (50.1)	224 (36.1)	24 (3.9)	0.028
Informal	10 (14.9)	42 (62.7)	15 (22.4)	0	
Household Structure/Type					
Female-centred	18 (13.8)	66 (50.8)	46 (35.4)	0	0.372
Male-centred	18 (12.8)	76 (53.9)	42 (29.8)	5 (3.5)	
Nuclear	36 (9.3)	194 (50.0)	139 (35.8)	19 (4.9)	
Extended	4 (7.7)	29 (55.8)	17 (32.7)	2 (3.8)	
Main Source of HH Income					
Formal wage work	16 (5.0)	174 (54.9)	110 (34.7)	17 (5.4)	<0.001
Informal wage work	45 (20.0)	115 (51.1)	62 (27.6)	3 (1.3)	
Self-employment (Informal)	5 (5.7)	31 (35.6)	46 (52.9)	5 (5.7)	
Self-employment (formal)	10 (12.7)	41 (51.9)	27 (34.2)	1 (1.3)	
Net Monthly HH Income					
KES ≤ 10,000	34 (24.1)	73 (51.8)	33 (23.4)	1 (0.7)	<0.001
KES 10,001–20,000	10 (13.3)	41 (54.7)	23 (30.7)	1 (1.3)	
KES 20,001–30,000	2 (5.9)	16 (47.1)	14 (41.2)	2 (5.9)	
KES 30,001–40,000	2 (11.1)	10 (55.6)	4 (22.2)	2 (11.1)	
KES 40,001–50,000	1 (6.7)	6 (40.0)	7 (46.7)	1 (6.7)	
>KES 50,000	2 (3.1)	20 (31.3)	33 (51.6)	9 (14.1)	
% of HH Income Spent on Food					
<20%	21 (26.6)	35 (21.7)	23 (29.1)	0 (0.0)	0.005
21–35%	6 (9.8)	34 (55.7)	19 (31.1)	2 (3.3)	
36–50%	9 (12.7)	41 (57.7)	16 (22.5)	5 (7.0)	
>50%	13 (10.2)	51 (40.2)	55 (43.3)	8 (6.3)	
Lived Poverty Index Categories					
0–0.5	27 (5.9)	217 (47.3)	191 (41.6)	24 (5.2)	<0.001
0.51–1.00	19 (13.1)	91 (62.8)	33 (22.8)	2 (1.4)	
1.01–1.50	14 (24.1)	33 (56.9)	11 (19.0)	0	
>1.50	14 (40.0)	16 (45.7)	5 (14.3)	0	
Sent Remittances					
No	45 (11.7)	199 (51.7)	127 (33.0)	14 (3.6)	0.815
Yes	32 (10.3)	156 (50.0)	113 (36.2)	11 (3.5)	
Received Food Transfers from Rural Areas					
No	51 (11.6)	216 (49.0)	154 (34.9)	20 (4.5)	0.177
Yes	25 (9.4)	144 (54.3)	91 (34.3)	5 (1.9)	
HH Shares Meal with Neighbours					
No	69 (10.7)	328 (50.7)	228 (35.2)	22 (3.4)	0.661
Yes	8 (11.3)	38 (53.5)	21 (29.6)	4 (5.6)	
HH Purchase Food from Supermarket					
No	33 (18.8)	100 (56.8)	41 (23.3)	2 (1.1)	<0.001
Yes	44 (8.1)	226 (49.1)	208 (38.4)	24 (4.4)	
HH Engage in Urban Agriculture					
No	76 (10.8)	359 (51.2)	240 (34.2)	26 (3.7)	0.390
Yes	1 (5.9)	7 (41.2)	9 (52.9)	0	
Gone without Certain Foods due to Food Price Increase					
Never	11 (3.9)	134 (47.7)	120 (42.7)	16 (5.7)	<0.001
About once a month	13 (7.8)	75 (44.9)	74 (44.3)	5 (3.0)	
About once a week	19 (14.8)	74 (57.8)	31 (24.2)	4 (3.1)	
>Once a week to every day of the week	32 (23.4)	80 (58.4)	24 (17.5)	1 (0.7)	
Gone without Cereals/Grains due to Price Increase					
Yes	41 (7.9)	255 (49.2)	202 (39.0)	20 (3.9)	<0.001
No	36 (18.0)	111 (55.5)	47 (23.5)	6 (3.0)	
Gone without Roots and Tubers due to Price Increase					
Yes	62 (9.8)	329 (52.0)	219 (34.6)	23 (3.6)	0.147
No	15 (17.6)	37 (43.5)	30 (35.3)	3 (3.5)	
Gone without Vegetables due to Price Increase					
Yes	69 (10.5)	346 (52.6)	220 (33.4)	23 (3.5)	0.041
No	8 (13.3)	20 (33.3)	29 (48.3)	3 (5.0)	
Gone without Fruits due to Price Increase					
Yes	64 (10.0)	333 (52.0)	222 (34.6)	22 (3.4)	0.190
No	13 (16.9)	33 (42.9)	27 (35.1)	4 (5.2)	
Gone without Meat, Poultry, or Offal due to Price Increase					
Yes	23 (5.4)	209 (49.4)	171 (40.4)	20 (4.7)	<0.001
No	54 (18.3)	157 (53.2)	78 (26.4)	6 (2.0)	
Gone without Eggs due to Price Increase					
Yes	59 (8.9)	340 (51.4)	237 (35.9)	25 (3.8)	<0.001
No	18 (31.6)	26 (45.6)	12 (21.1)	1 (1.8)	
Gone without Fish and Seafood due to Price Increase					
Yes	36 (7.3)	259 (52.2)	179 (36.1)	22 (4.4)	<0.001
No	41 (18.5)	107 (48.2)	70 (31.5)	4 (1.8)	
Gone without Pulses/Legumes/Nuts due to Price Increase					
Yes	68 (10.1)	345 (51.0)	239 (35.4)	24 (3.6)	0.091
No	9 (21.4)	21 (50.0)	10 (23.8)	2 (4.8)	
Gone without Milk and Milk Products due to Price Increase					
Yes	51 (8.4)	316 (52.0)	219 (36.0)	22 (3.6)	<0.001
No	26 (23.6)	50 (45.5)	30 (27.3)	4 (3.6)	
Gone without Oils/Fats due to Price Increase					
Yes	69 (10.6)	331 (50.9)	228 (35.1)	22 (3.4)	0.693
No	8 (11.8)	35 (51.5)	21 (30.9)	4 (5.9)	
Gone without Sugar/Honey due to Price Increase					
Yes	60 (9.5)	322 (51.1)	226 (35.9)	22 (3.5)	0.026
No	17 (19.3)	44 (50.0)	23 (26.1)	4 (4.5)	

**Table 8:** Ordinal logistic regression of determinants of household diet diversity.

Migrant Household Characteristics	OR (95% CI) *
Sex of Household Head	1.203 (0.669–2.163)
Age of Household Head	1.085 (0.923–1.274)
Education Level of Household Head	1.535 (1.212–1.944) ***
Employment Status of Household Head	0.770 (0.646–0.919) ***
Duration of Stay in Nairobi City	1.001 (0.884–1.133)
Health Status of HH head	1.216 (0.448–3.302)
Household Characteristics	
Household Size	0.978 (0.827–1.157)
Illness in HH	0.884 (0.538–1.450)
Dwelling Type	1.273 (0.825–1.966)
Household Structure/Type	0.942 (0.706–1.257)
Main Source of HH Income	1.054 (0.898–1.237)
Net Monthly HH Income	1.108 (0.983–1.249) *
% of HH Income Spent on Food	0.977 (0.835–1.143)
Lived Poverty Index Categories	0.792 (0.647–0.970) **
Sent Remittances	1.150 (0.843–1.569)
Received Food Transfers from Rural Areas	0.986 (0.653–1.488)
HH Share Meal with Neighbours	0.895 (0.544–1.474)
HH Purchase Food from Supermarket	1.235 (0.850–1.793)
HH Engages in Urban Agriculture	0.927 (0.295–2.913)
HFIAP	0.760 (0.630–0.917) ***
Gone without Certain Foods due to Food Price Change	0.941 (0.763–1.161)
Increase in Price of Cereals/Grains	0.988 (0.736–1.609)
Increase in Price of Roots and Tubers	0.832 (0.479–1.445)
Increase in Price of Vegetables	1.790 (1.001–3.340) **
Increase in Price of Fruits	2.098 (1.238–3.555) ***
Increase in Price of Meat, Poultry, or Offal	0.912 (0.594–1.399)
Increase in Price of Eggs	0.608 (0.316–1.173)
Increase in Price of Fish and Seafood	0.989 (0.638–1.957)
Increase in Price of Pulses/Legumes/Nuts	1.025 (0.524–2.006)
Increase in Price of Milk and Milk Products	0.817 (0.484–1.378)
Increase in price of Oil/Fat	1.261 (0.711–2.236)
Increase in Price of Sugar/Honey	0.888 (0.495–1.593)

Notes: OR = Odds Ratio; CI = Confidence Interval; * *p* ≤ 0.1, ** *p* ≤ 0.05, *** *p* ≤ 0.01.

## Data Availability

The data is held in a public repository at: https://www.datafirst.uct.ac.za/dataportal/index.php/catalog/843.
